# Recent Advances: Molecular Mechanism of RNA Oxidation and Its Role in Various Diseases

**DOI:** 10.3389/fmolb.2020.00184

**Published:** 2020-07-31

**Authors:** Zhe Li, Xiatian Chen, Ziqian Liu, Wei Ye, Ling Li, Lili Qian, Hongyan Ding, Peifeng Li, Lynn Htet Htet Aung

**Affiliations:** ^1^Center for Molecular Genetics, Institute for Translational Medicine, The Affiliated Hospital of Qingdao University, College of Medicine, Qingdao University, Qingdao, China; ^2^School of Basic Medicine, Qingdao University, Qingdao, China; ^3^Jiangsu Provincial Engineering Research Center for Biomedical Materials and Advanced Medical Device, Huaiyin Institute of Technology, Huaian, China

**Keywords:** RNA oxidation, oxidative damage mechanism, 8-hydroxyguanosine (8-OHG), disease, research progress

## Abstract

Compared with the research on DNA damage, there are fewer studies on RNA damage, and the damage mechanism remains mostly unknown. Recent studies have shown that RNA is more vulnerable to damage than DNA when the cells are exposed to endogenous and exogenous insults. RNA injury may participate in a variety of disease occurrence and development. RNA not only has important catalytic functions and other housekeeping functions, it also plays a decisive role in the translation of genetic information and protein biosynthesis. Various kinds of stressors, such as ultraviolet, reactive oxygen species and nitrogen, can cause damage to RNA. It may involve in the development and progression of diseases. In this review, we focused on the relationship between the RNA damage and disease as well as the research progress on the mechanism of RNA damage, which is of great significance for the pathogenesis, diagnosis, and treatment of related diseases.

## Introduction

As the main component of genetic material, DNA damage and repair mechanism has been paid close attention by researchers. However, RNA damage has not been paid enough attention resulting in an underestimated toxic effect of RNA damage on cells and the body. In a cell, RNAs are more abundant than DNAs, accounting for 80–90% of the cell’s total nucleic acid. Antisense RNA can bind to the target site by complementing the target site sequence, or directly prevent its function or change the conformation of the target site to affect its function. DNA post-transcriptional processing and modification usually involve some special RNA. RNA plays an important role in biological evolution. The discovery of ribozyme indicates that RNA is both an information molecule and a functional molecule, and RNA may be the first to appear in the early origin of life (Higgs and Lehman, 2015). Studies have shown that RNA damage exists in both physiological and pathological conditions (Ishii and Sekiguchi, 2019). A large number of experiments have shown that when RNA damage is severe, it may affect its normal physiological function and damage cells and organisms (Yan and Zaher, 2019; Giorgio et al., 2020). Since most DNA is double-stranded and the nucleotide bases on the two strands complement each other, bases are protected by hydrogen bonds and their structure is relatively stable and less subject to degradation (Vologodskii, 2016), while RNA is generally single-stranded and its structure is unstable (Sankaran, 2016). RNA instability may also be related to the half-life of the mRNA. Of the three common RNAs, the one with the shortest half-life is mRNA, which is immediately degraded after translation and is therefore the most unstable. tRNA and rRNA are relatively stable and generally exist for a long time (Peterson et al., 2016; Mauger et al., 2019). RNA is more abundant in cytoplasm and located near mitochondria at the subcellular level, and the bases are not protected by hydrogen bonds, which makes RNA more vulnerable to damage than DNA under the same nucleic acid damage pressure (Hofer et al., 2006; Nunomura et al., 2012; Fimognari, 2015).

Various kinds of stress, such as ultraviolet (UV), reactive oxygen species (ROS) and reactive nitrogen species (RNS), can cause damage to RNAs (Wurtmann and Wolin, 2009). RNA damage may have serious detrimental effects on the multifaceted functions of RNA and the viability of cells or organisms. Oxidative damage of ROS or RNS is a common damage in cells and can affect all macromolecules under physiological and pathological conditions (Li et al., 2014). Oxidized RNAs can be detected in rats, human urine and human plasma under normal physiological conditions. In particular, significant RNA oxidative damage can be detected in aging animal models and neurons of patients with neurodegenerative diseases (Nunomura, 2013). ROS are the main cause of RNA oxidative damage which are generated through the Fenton reaction (iron catalyzed oxidation) and are promoted by mitochondrial dysfunction *in vivo* (Poulsen et al., 2012). Ultraviolet radiation in the environment can cause photochemical modification and cross-linking of RNAs and can also cause RNA damage. Nitric oxide produced by the inflammatory response *in vivo* can also damage RNAs (Ishii et al., 2005).

At present, although there is an increasing number of studies on oxidative damage of RNA, the mechanism is still unclear. The purpose of this review was to describe the relationship between the RNA damage and disease as well as the research progress on the mechanism of RNA damage, which is of great significance for the pathogenesis, diagnosis, and treatment of related diseases.

## Research Progress on Oxidative Damage Mechanism of RNA

At present, it is believed that cells have corresponding repair mechanisms for different forms of RNA oxidative damage. However, it is not still clear whether cells have corresponding repair mechanisms for oxidative damage of RNA, but some studies have shown that stress molecules involved in DNA damage may participate in oxidative damage stress response of RNAs (Mikolaskova et al., 2018). The mRNA monitoring and control mechanisms can remove the wrong mRNA transcripts, including nonsense codon mediated mRNA degradation (NMD), mRNA degradation without termination codon, and degradation of translation blocked mRNA, which may be involved in the treatment of mRNA damage. Protein truncations translated from oxidized mRNA may activate the NMD process (Fukuhara et al., 2005; Sheth and Parker, 2006). The repair mechanism of RNA methylation damage is the same as that of DNA damage. AlkB in *E. coli* and homologous molecule hABH3 in mammals can reverse the damage by direct hydroxylation of methyl groups on DNA or RNA bases damaged by methylation (Liu et al., 2010; Boniecki and Martinis, 2012). The most prevalent oxidized base in RNA is 8-hydroxyguanosine (8-OHG), which is oxidized by hydroxyl radicals to form C8-OH adjunct radicals. Different studies demonstrate that the oxidation of RNA can lead to the breaking of the stranded and the modification or removal of the bases (Nunomura et al., 2009; Jacobs et al., 2010).

**FIGURE 1:**
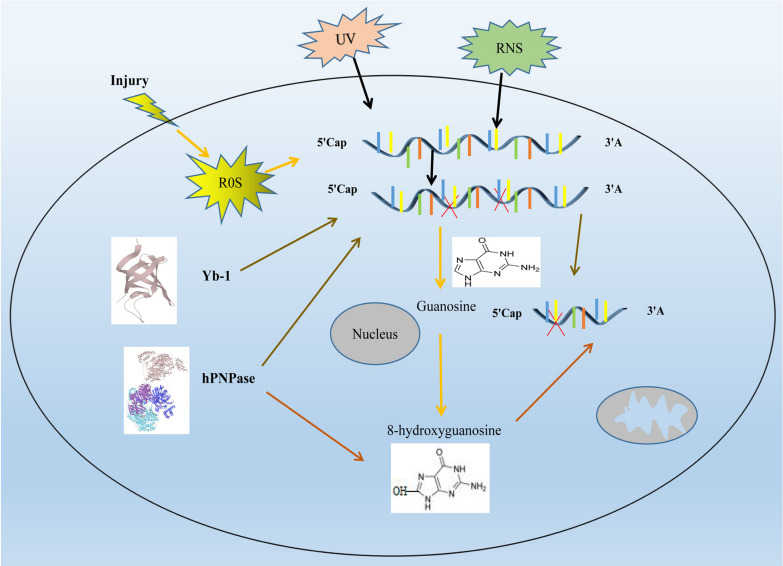
Research progress on oxidative damage mechanism of RNA. Ultraviolet (UV), reactive oxygen species (ROS) and reactive nitrogen species (RNS), will cause damage to RNAs RNA oxidative damage in cell. The increase of intracellular ROS leads to oxidative damage to RNA, which leads to an increase in intracellular 8-hydroxyguanosine (8-OHG), it is formed because the guanosine in RNA in RNA can be oxidized by highly reactive hydroxyl radicals to form a C8-OH adduct radical. hPNPase can degrades damaged RNA and reduces the content of 8-OHG, thereby reducing RNA damage. Yb-1 attenuates RNA damage by silencing damaged RNA. RNA molecules are shown as blue lines. red X represent the damaged RNA.

8-OHG has been the focus of studies because it appears to be mutagenic (Castellani et al., 2008). For example, *E. coli* neutralized polynucleotide phosphorylase (hPNPase), human cell y-box binding protein 1 (Yb-1) and heterogeneous nuclear ribonucleoprotein (hnRNP) in human cells, which may lead to the silencing of damaged RNA and thus terminate its translation process after binding to damaged RNA (Maughan and Shuman, 2015). hPNPase is a human 3′-5′ RNA exonuclease that specifically degrades damaged RNA, update coding and non-coding RNA under the action of various damage recognition proteins. Overexpression of hPNPase can reduce the content of 8-OHG and improve the cell viability under oxidative stress. After hPNPase knockdown, the content of oxidative damaged RNA increases and the cell viability decreases (Wu and Li, 2008). Yb-1 is a component of intracellular mRNA degradation complex p-body. In the process of oxidative stress, Yb-1 may be involved in mediating the degradation of damaged RNAs (Figure 1). After interference with HNRNP, the sensitivity of cells to H_2_O_2_ stimulation was enhanced (Das et al., 2007; Hayakawa et al., 2010).

Some studies have shown that capillary dilated ataxia mutant protein (ATM), may be involved in RNA damage stress response. ATM is an important DNA damage stress molecule, and the autophosphorylation at serine is a marker of its activation. The activity and signaling pathway of ATM are disrupted after the mutation of the self-phosphorylation site (Bakkenist and Kastan, 2003). Recent studies reported that ATM participates in cellular RNA oxidative damage stress through autophosphorylation activation (Bhatia et al., 2018). However, the role of ATM in RNA damage stress, as well as its involvement in RNA damage recognition, repair, or degradation and renewal, are yet to be explored. Furthermore, studies of animal and human models suggest that the role of modified ribonucleotides in cells may be more profound. For example, m6A has been reported to be involved in transcriptional splicing and transposition in addition to regulating mRNA stability. Studies on leukemia cells have shown that complexes formed by m5C and specific proteins can regulate chromatin structure and its affinity to biomolecules (Chmielowska-Bąk et al., 2019).

Unlike AUF1, which recognizes an 8-OHG RNA, PCBP1 recognizes heavily oxidized RNA. This factor does not promote the degradation of target cells but induces cell death. A mutation occurs in one of the two RNA binding zinc finger domain and hnRNPK-homology domain (KH) domains cancel the binding of 8-OHG and inhibit the induced RNA binding activity of apoptosis-related reactions, which is necessary to induce programmed cell death. These observations underscore the ability of cells to use damaged mRNA as a signal of stress. Moreover, this PCBP1-mediated response is activated only in the presence of a large amount of modified RNAs (Ishii et al., 2018), which are activated only in the presence of excessive oxidative stress (Table 1).

**TABLE 1:** Summary the research progress of RNA oxidative damage mechanism.

Condition	Observed in	Finding	References
ATM	Mammalian cells	ATM involved in RNA damage stress response	Bakkenist and Kastan, 2003
mRNA damage	Metazoa/yeast	Protein truncations translated from oxidized mRNA may activate the NMD process	Fukuhara et al., 2005; Sheth and Parker, 2006
Yb-1	HeLa cell/Mammalian cells	Yb-1 involved in mediating the degradation of damaged RNA	Das et al., 2007; Hayakawa et al., 2010
hPNPase	Escherichia coli/HeLa cell	hPNPase lead to the silencing of damaged RNA and thus terminate its translation process after binding to damaged RNA	Wu and Li, 2008; Maughan and Shuman, 2015
The product of oxidative damage to RNA	Mammalian cell	The most prevalent oxidized base in RNA is 8-OHG	Nunomura et al., 2009; Jacobs et al., 2010
PCBP1	HeLa S3 line	PCBP1 recognizes heavily oxidized RNA and induces cell death	Ishii et al., 2018

## Relationship Between Oxidative Damage of RNA and Diseases

**FIGURE 2:**
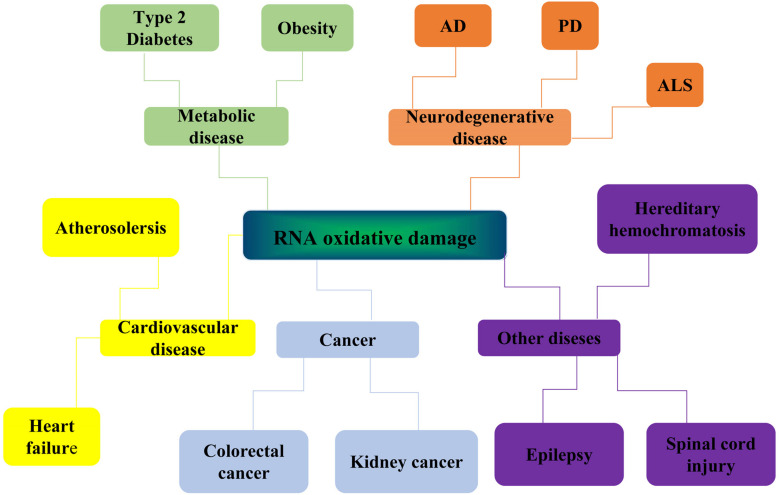
Diseases related to RNA oxidative damage. RNA oxidative damage is associated with the development of many diseases. It is mainly related to the occurrence and development of four kinds of diseases: neurodegenerative diseases, metabolic diseases, cardiovascular diseases, and cancer. Neurodegenerative diseases include Alzheimer’s disease (Alzheimer diseases, AD), Parkinson’s disease (PD) and Amyotrophic lateral sclerosis (ALS). Metabolic diseases include type 2 diabetes and obesity. Cardiovascular diseases include atherosclerosis and heart failure. Cancer include colorectal cancer and kidney cancer. It is also associated with hereditary hemochromatosis, spinal cord injury, epilepsy, and other diseases.

The damage of genomic coding RNAs can affect gene expression and cause the disorder of proteome expression. About 98% of gene transcripts are non-coding RNAs. Studies have shown that non-coding RNAs have structural functions and catalytic activities. In recent years, a large number of non-coding RNAs have been discovered and identified in the central nervous system. Therefore, the physiological dysfunction caused by RNA damage has a close and complex relationship with the pathophysiology of neurons. Recently, more and more experiments have proved that oxidative RNA damage is related to the pathogenesis of neurodegenerative diseases. Obvious oxidative RNA damage can be detected in vulnerable neurons in the early stage of neurodegenerative disease, indicating that RNA oxidation may contribute to the occurrence and development of the disease (Butterfield et al., 2001). In addition, RNA damage may also be associated with atherosclerosis, type 2 diabetes, cancers, and other diseases (Figure 2).

### Neurodegenerative Disease

Studies found that patients at the early and middle phase of Alzheimer’s disease (Alzheimer diseases, AD) showed up to 50% of mRNA in their frontal cortex had oxidative damage, but in the control group mRNA damage degree is lower than 2%, and further combining with DNA chip analysis found that many mRNAs are associated with the AD (Shan et al., 2003; Ahmad et al., 2017). It is worth noting that the AD’s and mild cognitive impairment (MCI) patients, the rate of protein synthesis and ribosome complex ability in brain tissue specific areas are significantly drop. More interestingly, the ribosome concentration of rRNA and tRNA decreased in this areas, while the content of 8-OHG increased, suggesting the dysfunction of ribosome related to RNA oxidative damage (Ding et al., 2006).

In addition, several studies also detected a significant increase in RNA oxidative damage in Parkinson’s disease (PD) (Yoritaka et al., 1996; Ding et al., 2005; Annesley et al., 2016). Elevated RNA oxidation along with the oxidative damage to lipid, protein, and DNA were also observed in postmortem substantia nigra tissue and Cerebrospinal Fluid (CSF) from living PD patients (Zhang et al., 1999; Kikuchi et al., 2002; Abe et al., 2003). Investigations of the relationship between the levels of 8-OHG in the CSF and the duration of disease suggest that RNA oxidation may occur in the early stage of PD. However, studies are needed to determine if RNA oxidation contributes to the pathogenesis of PD.

Amyotrophic lateral sclerosis (ALS) is one of the most devastating neurological disorders (Kuzma-Kozakiewicz and Kwiecinski, 2011). Study suggest that oxidative stress can modulate the function of membrane related receptor protein tyrosine kinase c-ret, thereby reducing glial cell line-derived neurotrophic factor (GDNF) signaling in motor neurons (Ryu et al., 2011).

### Cardiovascular Disease

It has also been reported that the intracellular contents of 18 s and 28 s rRNA in atherosclerotic patients decrease with the increase of 8-OHG (Li et al., 2019). Oxidative stress plays a key role in the progression of atherosclerosis. Elevated oxidative damage to DNA has been reported in both human and experimental atherosclerosis (Martinet et al., 2001; Tu et al., 2019). All RNA oxidation had been detected in smooth muscle cells and endothelial cells of the human atherosclerotic plaques (Martinet et al., 2004, 2005). The implications of RNA oxidation in atherosclerosis still remain unknown. Studies have found that RNA oxidation is associated with the occurrence and development of a variety of chronic diseases in the elderly, but whether RNA oxidation is related to the pathogenesis of HF remains unclear (Liu et al., 2019).

### Metabolic Disease

Recent studies have shown that levels of RNA oxidative damage in the urine of patients with type 2 diabetes can predict long-term mortality. The urinary excretion of the RNA oxidation marker 8-OHG measured shortly after diagnosis of type 2 diabetes predicts the long-term mortality independently of other risk factors. Broedbaek et al. (2011) suggested that 8-OHG could serve as a new clinical biomarker in diabetes (Kjær et al., 2017). There are studies found high RNA oxidation is associated with an cause and cardiovascular mortality risk in patients with type 2 diabetes (Rafeh et al., 2020; Whipple et al., 2020; Yandrapalli et al., 2020). One study examined the urine content of 8-OHG in obese normal men, thin normal men and normal weight men, and found that the urine content of 8-OHG in obese normal men was higher than that in other groups, indicating that obesity in men is associated with increased RNA oxidative damage (Cejvanovic et al., 2016). These results suggest that RNA oxidation may be involved in the pathophysiological process of chronic diseases.

### Cancer

Besides, oxidative damage to RNA may also be related to the development of cancers. Compared to normal cells, cancer cells exhibit an accelerated metabolism and demand high ROS concentrations to maintain their high proliferation rate. ROS is of high significance in cancer treatment and it influences tumor outcome (Andrisic et al., 2018). Recent research has found that 8-OHG as a possible RNA oxidative modification marker in urine from colorectal cancer patients associated lipid peroxidation might eventually help to defend adjacent non-malignant cells from cancer invasion (Guo et al., 2020). The number of kidney cancer is growing 3–5% each year due to unknown etiologies. Intra and inter-tumor mediators increase oxidative stress and drive tumor heterogeneity. Clinical analyses of large renal tumors reveal substantial heterogeneity within the tumors themselves, which are composed of low and high-grade tumor regions with areas of necrosis and vascularity. This heterogeneity likely explains drug resistance, despite targeted therapies. ROS play a major role in the development and progression of renal carcinogenesis (Shanmugasundaram and Block, 2016; Lichner et al., 2019). Studies have found that changes in RNA damage can promote the occurrence of tumors (Jorgensen et al., 2018; Janostiak and Wajapeyee, 2019).

### Other Diseases

Investigated RNA oxidation after spinal cord contusion injury in rats. There are findings a significant increase in RNA oxidation in the lesion center immediately after the injury and the level remains high over 3 h, while in the cord segment rostral to the lesion center, RNA oxidation did not increase significantly until 3 h after the injury. Increased RNA oxidation primarily occurs in the neurons around the lesion center at an early stage and in the oligodendrocytes during secondary injury progression, and these cells die later. These results indicate a spreading pattern of the RNA oxidation from the lesion site to the distal areas, and this coincides with the developmental pattern of the secondary injury. These results suggest that RNA oxidation may play an important role in the development of secondary injury (Xu et al., 2005; Kong and Lin, 2010).

As a common chronic neurological disorder, epilepsy is characterized by recurrent unprovoked seizures. It afflicts more than 50 million people worldwide. Studies have demonstrated that seizure-induced mitochondrial dysfunction and excess free radical production cause oxidative damage to cellular components and initiate the mitochondrial apoptotic pathway (Henshall et al., 2001, 2002).

Oxidatively generated damage to nucleic acids is considered to play a significant role in carcinogenesis, and it has been shown that people with hereditary hemochromatosis are at increased risk of cancer. In this study, they used a new refined liquid chromatography-tandem mass spectrometry method to measure the level of 8-oxo-7 and 8-OHG in the urine of hereditary hemochromatosis patients, and they investigated the effect of treatment on the levels of these modifications. The study was carried out as a classical case-control study of 21 newly diagnosed, never treated hereditary hemochromatosis patients and 21 matched controls. They found that the levels of 8-OHG in the urine of the experimental group were much higher than those of the control group, with a similar level of 8-oxo-7, which returned to normal after treatment (Broedbaek et al., 2009; Lawrence et al., 2018). 8-OHG is the signature product of RNA oxidative damage, it can be seen that hereditary hemochromatosis is related to the oxidative damage of RNA.

## Conclusion and Perspectives

At present, the research on the mechanisms of RNA damage oxidation is still scarce. RNA oxidative damage is involved in the pathogenesis of various pathological diseases, such as ischemia, cancer, obesity, diabetes, viral infections, chronic fatigue syndrome, amyotrophic lateral sclerosis (ALS), depression, kidney disease, heart failure, and atherosclerosis. RNA oxidation is a marker of many neurodegenerative diseases as well as an indicator of an increase in oxidative stress which can lead to an exacerbation of diseases. However, the molecular mechanism of RNA oxidative damage in these diseases remains to be explored, the direct cause-and-effect relationship between oxidized RNA and disease pathogenicity is not clear (Simms and Zaher, 2016). Although different forms of RNA damage are known to trigger different mechanisms of recognition and repair, whether there is a common pathway between multiple complex stress responses, whether RNA damage and DNA damage share some signaling pathways will be the focus of our future.

## Author Contributions

ZhL, LA, and PL discussed the structure and content of the review. ZhL wrote the manuscript. LA and PL revised the manuscript. XC and ZiL helped to revise the manuscript. WY, LL, LQ, and HD did the final editing. All authors contributed to the article and approved the submitted version.

## Conflict of Interest

The authors declare that the research was conducted in the absence of any commercial or financial relationships that could be construed as a potential conflict of interest.
